# App-Based Stabilization Phase Within Blended Trauma-Focused Cognitive Behavioral Therapy With Compassion for Adolescents With Posttraumatic Stress Disorder: Formative Qualitative Study

**DOI:** 10.2196/95633

**Published:** 2026-10-15

**Authors:** Linda Wallin, Nick Nilsson, Inga Dennhag

**Affiliations:** 1 Child and Adolescent Psychiatry, Department of Clinical Science, Umeå University, Akutvägen 4C, Building 28, NUS, Umeå, 90187, Sweden, +46 70 210 17 26

**Keywords:** digital health, posttraumatic stress disorder, PTSD, adolescents, stabilization phase, compassion focused therapy, qualitative formative research

## Abstract

**Background:**

Digital tools may support early phases of trauma therapy for adolescents, yet little is known about how an app-based stabilization phase functions in routine child and adolescent psychiatry clinics. To our knowledge, no previous study has qualitatively examined a compassion-integrated, app-based stabilization phase delivered before trauma processing within trauma-focused cognitive behavioral therapy (TF-CBT) for adolescents.

**Objective:**

This formative qualitative pretrial study explored the acceptability, feasibility, and contextual fit of an app-based stabilization phase within blended TF-CBT with compassion (bTF-CBT-C) for adolescents with posttraumatic stress disorder (PTSD). The aim was to inform the refinement of intervention content, caregiver support, and clinical workflow prior to a planned pilot randomized controlled trial (RCT).

**Methods:**

Four adolescent girls with PTSD (aged 14‐16 years) completed a 5-week app-based stabilization program integrating TF-CBT coping skills and compassion-focused practices, while 5 caregivers received parallel content in a nonapp format. Three adolescents and 4 caregivers participated in postintervention semistructured interviews, and 4 clinicians completed a brief open-ended survey. Data were analyzed using reflexive thematic analysis.

**Results:**

One overarching theme, *“*Navigating digital stabilization within shifting conditions,” captured how engagement fluctuated with emotional capacity, cognitive load, relational dynamics, and logistical stability. Adolescents and caregivers experienced the intervention content as grounding and meaningful but difficult to sustain amid fatigue, school pressure, neurodivergent attention patterns, and family strain. Clinicians similarly noted varying capacity, the need for scaffolding, and the importance of reminders and smooth access. Clear content could exceed users’ cognitive bandwidth; adolescents preferred autonomy, while caregivers were unsure how to support them; and technical friction sometimes disrupted momentum. Across the groups, participants described grounding and compassion-based practices as supporting emotional safety, shared understanding, and readiness for trauma-focused work.

**Conclusions:**

The findings suggest that app-based stabilization within bTF-CBT-C may be acceptable and potentially feasible for adolescents with PTSD, their caregivers, and clinicians. Feasibility appears to depend on flexible pacing, cognitive accessibility, caregiver guidance, and technical reliability. The study identifies candidate refinements to be evaluated in the pilot RCT and provides preliminary insight into how compassion-integrated digital stabilization may support preparation for trauma-focused therapy.

## Introduction

### Background

Digital tools are increasingly used to support psychological treatment for adolescents [1,2], yet relatively little is known about how these tools function in the realities of adolescents' everyday lives. Digital formats can lower barriers related to travel, scheduling, and clinician availability, challenges that are particularly pronounced in rural regions [3]. At the same time, studies indicate that adolescents’ engagement with digital interventions is often shaped by day-to-day variations in energy, attention, school pressure, and emotional capacity [1,2]. These shifting conditions highlight the importance of flexible and accessible formats during the early stages of therapy, when stability and repeated practice of regulation skills are central. This is particularly relevant in trauma-focused treatments, where the stabilization phase serves as the initial foundation for later therapeutic work [4]. Given that little is known about how adolescents engage with stabilization work in digital formats, a formative study is needed to understand how an app-based approach functions in practice; what works, what does not, and what requires refinement before a pilot randomized controlled trial (RCT).

Exposure to potentially traumatic events is common among adolescents, with prevalence estimates indicating that up to 80% of adolescents experience at least 1 potentially traumatic event [5], and approximately 20% to 25% of adolescents develop posttraumatic stress disorder (PTSD) [6-8]. Untreated PTSD is associated with substantial long-term consequences, including emotional instability, academic difficulties, social withdrawal, increased risk of substance misuse, suicidality, and elevated psychiatric comorbidity [9-13]. Without timely and developmentally attuned intervention, these difficulties may persist in adulthood and affect relational, occupational, and overall functioning.

Trauma-focused cognitive behavioral therapy (TF-CBT) is the recommended first-line treatment for adolescent PTSD and is typically delivered across 3 phases: stabilization, trauma narration, and integration [14-16]. Despite a strong evidence base, implementing the model in routine care can be challenging. Many families struggle with between-session practice, caregiver availability varies, and emotional demands can make it difficult for adolescents to maintain consistent engagement [17,18]. These challenges are amplified in rural contexts where services span large geographical areas and access to trained clinicians is uneven [3,19,20].

Although stabilization is widely regarded as a crucial first phase of TF-CBT, this component has rarely been studied as a distinct therapeutic process. Research has primarily evaluated full TF-CBT protocols, whereas empirical work examining digital or preparatory emotion-regulation interventions for adolescents remains limited. Blended formats, where app-based exercises complement therapist-led sessions, may help address these gaps [21]. Digital components can provide flexibility, continuity, and short, manageable learning units suited to adolescents’ fluctuating emotional and cognitive capacities [2,22-24]. They may also support self-regulation between sessions, an area families consistently describe as difficult in routine care. These issues are particularly relevant in northern Sweden, where child and adolescent psychiatry services must operate across vast geographical areas with long travel distances and uneven specialist availability. Prior qualitative work from the region indicates that adolescents themselves express a clear preference for individualized, blended formats that can support continuity and reduce barriers to trauma therapy [25]. Integrating digital materials early in treatment may therefore strengthen treatment accessibility, readiness, and stability before entering trauma-focused work.

Compassion-focused strategies derived from compassion-focused therapy (CFT) may further strengthen the stabilization phase by addressing mechanisms that commonly hinder engagement in trauma-focused work among adolescents, such as shame, self-criticism, and persistent activation of the threat system [26-28]. Compassion practices are designed to cultivate emotional safety and self-soothing, which can increase adolescents’ tolerance for distress and facilitate an approach toward difficult memories and emotions [27,29,30]. Preliminary work suggests that compassion-based exercises may translate effectively to digital formats, offering grounding, support, and affect-regulatory scaffolding during emotionally demanding periods [30-33]. Compassion strategies may also enhance coregulation within caregiver-youth relationships, helping caregivers respond with greater attunement and reducing relational patterns that sustain avoidance or emotional dysregulation [29,34]. Because digital stabilization work unfolds across adolescents’ daily routines, caregiver involvement, and clinical workflows, multistakeholder perspectives are essential for determining whether an app-based stabilization phase is feasible and acceptable in real-world practice.

Although digital interventions have shown promise across a range of adolescent mental health difficulties, including anxiety and depression [1,2], research on app-based or blended trauma-focused treatments specifically for adolescents with PTSD remains scarce. Recent work has explored digital tools that complement standard TF-CBT, such as an app designed to support psychoeducation, coping, and between-session practice for adolescents [35]. However, these tools have primarily functioned as adjunctive support to standard treatment rather than as structured, clinician-supported stabilization phases delivered before trauma processing.

To our knowledge, no previous study has qualitatively examined a structured, clinician-supported, compassion-integrated, app-based stabilization phase delivered before trauma processing within TF-CBT for adolescents.

Furthermore, little qualitative research has examined adolescents’, caregivers’, and clinicians’ experiences of such interventions. Most existing studies rely on quantitative designs, adult samples, or single-stakeholder perspectives [36]. To our knowledge, no formative qualitative work has explored how digital stabilization components function within the service conditions of northern Sweden, where geographical distance and service variability shape treatment delivery.

Taken together, these gaps highlight the need for early, multistakeholder formative work to examine the acceptability, feasibility, and contextual fit of an app-based, compassion-integrated stabilization phase prior to a pilot RCT. Formative evaluation is essential to ensure that content, usability, safety routines, and clinical workflows align with adolescents’ living conditions and with the realities of routine child and adolescent psychiatry (CAP) practice.

### Aim and Research Questions

This formative qualitative pretrial study aimed to examine the acceptability, feasibility, and contextual fit of a 5-week app-based stabilization phase within blended TF-CBT with compassion (bTF-CBT-C) for adolescents with PTSD. By integrating perspectives from adolescents, caregivers, and clinicians, the study aimed to identify concrete refinements to intervention content, caregiver involvement, usability, and clinical workflow prior to a planned pilot RCT.

The study addressed the following research questions:

1. How do adolescents, caregivers, and clinicians experience the acceptability and feasibility of the app-based stabilization phase within bTF-CBT-C?
2. What refinements to the intervention content, caregiver involvement, and clinical workflow are needed before the implementation of the pilot RCT?

## Methods

### Overview

This study used a formative qualitative pretrial design to explore how adolescents, caregivers, and clinicians experienced the 5-week app-based stabilization phase of bTF-CBT-C in routine CAP care. This approach aimed to obtain in-depth, multistakeholder insights to guide the refinement of the stabilization component before a planned pilot RCT. Formative evaluation assesses whether an intervention is acceptable, feasible, and appropriate for end users before scaling up and is commonly recommended as a preparatory step before later-stage trials. The study followed a user-centered design approach informed by the person-based approach, focusing on adolescents’, caregivers’, and clinicians’ lived experiences to guide the refinement of the intervention before pilot testing [20,37]. The person-based approach emphasizes grounding early-stage design decisions in rich qualitative insights to optimize usability, acceptability, and safety, which aligns with the aims of this formative work. Reporting of this study was informed by the Reflexive Thematic Analysis Reporting Guidelines (RTARG) developed by Braun and Clarke [38]. A completed RTARG reporting document is provided as Checklist 1.

### Study Setting

This study was conducted in northern Sweden, specifically in the Västerbotten and Norrbotten regions. Norrbotten is Sweden’s largest county, covering approximately 25% of the country’s land area and home to about 250,000 people. Västerbotten is the second largest, with roughly 280,000 inhabitants [39]. Both regions are characterized by long geographical distances, low population density, and uneven access to specialized CAP services. Travel to CAP clinics may involve long distances, and specialist resources are concentrated in a small number of clinics. In total, 7 CAP clinics serve the two regions; however, participants in the present study were recruited from 1 clinic in each region (Umeå in Västerbotten and Luleå in Norrbotten). This setting is relevant for understanding how blended and digital components may support care delivery in sparsely populated health care systems.

### Methodological Approach

A reflexive thematic analysis (RTA) was conducted following Braun and Clarke’s 6-phase framework [40-42]. RTA was selected because it is ideal for exploratory and developmental research aimed at understanding experiences and generating insights to refine interventions. Considering the study’s formative goal, all qualitative data (adolescent interviews, caregiver interviews, and clinician survey responses) were analyzed within a single, integrated RTA to identify patterns across stakeholder groups while still focusing on group-specific details.

An abductive analytic approach guided the interpretation, enabling shifts between data-driven insights and relevant conceptual elements of the blended TF-CBT model. This facilitated a nuanced understanding of user needs and areas for improvement within the stabilization component.

### Recruitment and Participants

#### Overview

Participants included adolescents, their caregiving adults, and clinicians involved in delivering or supporting bTF-CBT-C. Since the study was formative, no predefined target sample size was established; recruitment continued until the research team judged that the intervention and postintervention interviews could be completed within the project timeline. Each group is described separately below.

#### Adolescents

Adolescents were recruited from CAP waiting lists in Västerbotten and Norrbotten between June and October 2025. All adolescents who met the predefined clinical eligibility criteria were invited to participate along with a nonperpetrating caregiver. Two adolescents were screened out for not meeting eligibility criteria, so the final sample includes all eligible patients approached during the recruitment period.

Eligible participants were adolescents with a *DSM-5* (*Diagnostic and Statistical Manual of Mental Disorders, Fifth Edition*) PTSD diagnosis, confirmed through the Mini International Neuropsychiatric Interview for Children and Adolescents [43] and diagnostic case conference. A Child and Adolescent Trauma Screen-2 total score above 25 was required [44]. Additional inclusion criteria required at least 1 nonperpetrating caregiver and the ability to complete the 5-week app-based stabilization phase of bTF-CBT-C. Exclusion criteria included psychosis, severe dissociation, acute suicidality, autism spectrum disorder, severe eating disorder, ongoing trauma exposure or unsafe living conditions, active psychotherapy, recent medication changes, or regular benzodiazepine use.

Of those eligible, 4 female adolescents (aged 14‐16 years) enrolled, and all completed the app-based stabilization phase. Three participated in the postintervention interview. All adolescents met criteria for PTSD, with Child and Adolescent Trauma Screen-2 total scores ranging from 26 to 45, indicating moderate-to-severe symptom levels. Reported traumatic events included sexual assault (in person or online) and domestic violence. All adolescents had at least 1 comorbid psychiatric diagnosis, including conditions such as dyslexia, attention-deficit/hyperactivity disorder, nonsevere eating disorders, depressive symptoms, or social anxiety. The Children’s Global Assessment Scale [45] ranged from 44 to 50, consistent with significant functional impairment. Travel times to their local CAP clinic (Umeå or Luleå) ranged from 20 to 50 minutes.

#### Caregivers

Caregiver participation was important given TF-CBT’s established emphasis on caregiver involvement and the stabilizing role caregivers often play in supporting adolescents’ emotion regulation and treatment engagement. Each adolescent participated together with at least 1 nonperpetrating caregiver. In total, 5 caregivers were included: 3 mothers and 2 fathers (aged 39‐48 years), all of whom were employed. Four caregivers participated in interviews; 1 did not. As adolescents’ primary support figures, caregivers provided insight into how family resources, emotional capacity, and daily-life demands shaped feasibility and engagement during the app-based stabilization phase. Their perspectives were essential for understanding how the intervention aligned with family routines, how they interpreted and supported their adolescent’s emotional responses, and which structural or relational factors affected participation.

#### Clinicians

Four clinicians participated in the implementation phase of the project, and all agreed to complete a brief, open-ended survey that captured implementation-level perspectives.

All were licensed psychologists or licensed psychotherapists working within CAP in Västerbotten or Norrbotten. Clinical experience ranged from approximately 3 years to more than 25 years, with most clinicians positioned toward the senior end of this range. Their professional backgrounds included psychology, psychotherapy, and social work, and all clinicians were formally trained in TF-CBT and compassion-focused approaches. All clinicians were women, reflecting the gender distribution within the participating CAP services.

Together, adolescents, caregivers, and clinicians made up the multistakeholder sample. This sample size was suitable for an exploratory, formative qualitative study focused on generating in-depth, diverse, and practical insights.

### Data Collection Procedure

#### Overview

All eligible families first received verbal information about the study from their local CAP clinician. They then received written study information through the secure national health care platform (1177.se). For the interview phase, researchers contacted participants by telephone to schedule a time that suited them. Written informed consent was obtained from all participants prior to data collection; for adolescents under 15 years of age, caregiver consent was obtained first, in accordance with Swedish ethical guidelines. Participation was voluntary, and families were informed that they could withdraw at any time without consequences for their ongoing or future care. All interviews were conducted by NN, a medical student and member of the research team. NN was not involved in participants’ clinical care or treatment. Participants were informed about the interviewer’s role in the research project before participating. LW and ID developed the interview guides and refined them through discussions within the research team before data collection.

#### Adolescent Interviews

Data collection took place after adolescents had completed the 5-week app-based stabilization phase. Semistructured interviews were conducted individually with adolescents via Microsoft Teams, using an interview guide tailored to their developmental level and focusing on their experiences with the app’s content, exercises, emotional reactions, daily use, therapeutic contact, and overall feasibility. Interviews lasted approximately 45 minutes. With permission, all interviews were audio-recorded by an external adapter (not Teams) and transcribed verbatim using Klang.ai.

#### Caregiver Interviews

Caregivers were interviewed separately from adolescents to allow them to speak freely about family routines, emotional capacity, and support needs without the adolescent present. Caregiver interviews were also conducted via Microsoft Teams, using a caregiver-specific semistructured interview guide, and lasted approximately 45 minutes. Interviews explored perceptions of feasibility, alignment with family routines, support needs, and observations of the adolescent’s engagement. All interviews were audio-recorded with consent and transcribed verbatim using Klang.ai.

#### Clinician Survey

To capture clinicians’ perspectives on feasibility, workflow, and implementation, 4 clinicians who delivered bTF-CBT-C completed a brief, open-ended online survey. The survey was distributed several weeks after adolescents completed the stabilization phase, allowing clinicians to reflect on logistical issues, safety routines, usability, and integration with CAP workflows. Clinician participation in the survey was entirely voluntary. Survey prompts addressed usability, clinical fit, workload, and recommendations for adjustments before the pilot RCT. Written responses were treated as qualitative textual data and analyzed alongside adolescent and caregiver materials to identify system-level considerations for refinement.

### Ethical Considerations

The Swedish Ethical Review Authority approved the study (Dnr 2024-07057-01 and 2025-04566-02), and it was conducted in accordance with the Declaration of Helsinki and national regulations for research involving minors and trauma-exposed populations. All data were deidentified during transcription and stored on secure institutional servers in compliance with General Data Protection Regulation (GDPR). The clinical team monitored participants’ well-being throughout the study and identified no safety concerns requiring intervention beyond routine care.

Adolescents received a 200 SEK (approximately US $20; SEK 1=US $0.1009 as of September 25, 2026) gift card for completing study assessments and an additional 200 SEK gift card for participating in the postintervention interview. Caregivers and clinicians received no financial compensation.

### Intervention: Blended Trauma-Focused Cognitive Behavioral Therapy with Compassion

#### Overview

bTF-CBT-C is an adaptation of TF-CBT designed to improve accessibility and feasibility for adolescents, especially in rural areas or when families need flexible formats. The model combines core TF-CBT elements [4] with compassion-focused therapeutic strategies [26] to enhance emotional regulation, decrease shame-based responses, and encourage coordinated caregiver involvement. bTF-CBT-C is delivered using a blended approach, combining face-to-face or videoconference sessions with digital components.

#### App-Based Stabilization Module

The stabilization module precedes the trauma-focused phase and prepares adolescents and caregivers emotionally and cognitively for narrative work. The module integrates TF-CBT psychoeducation and coping skills with compassion-focused exercises that target self-soothing, emotion regulation, and shame reduction. Compassion components are adapted from a previously evaluated CFT intervention shown to reduce stress, anxiety, and depressive symptoms among adolescents [33], as well as the Kindness workbook [46].

The module comprises 5 modules, each designed to be completed in about 1 week (≈2 h). Modules were intended to be completed sequentially at a pace of approximately 1 module per week. New modules were released according to the planned schedule, which prevented participants from completing all modules in a single week while still allowing flexibility in when within each week they completed the material. Each module includes psychoeducation, videos, interactive exercises, grounding tools, and compassion-based practices, with quizzes and progress indicators to enhance engagement. For an overview of chapter structure and content, see Table 1.

The structure supports flexible self-pacing and gradual skill development while maintaining emotional safety throughout the stabilization phase.

**Table 1.** Overview of the stabilization phase of blended trauma-focused cognitive behavioral therapy with compassion (bTF-CBT-C) for adolescents with posttraumatic stress disorder (PTSD) in a qualitative pretrial study conducted at 2 child and adolescent psychiatry (CAP) clinics in northern Sweden (June-October 2025)^a^.

Week	Module	Delivery mode	Clinician contact	Purpose of module
0	Introduction	App + clinic session	One 60- to 90-minute clinic session with adolescent and caregiver	Establish therapeutic alliance, clarify structure, and initiate safety planning
1	Trauma and compassion	App + videoconference	One 45‐ to 60-minute videoconference session with adolescent and caregiver	Promote shared understanding of trauma and compassion; reduce shame through psychoeducation
2	Well-being and compassion with the body	App	Clinician support as needed	Activate the soothing system and increase bodily awareness for emotion regulation
3	Emotions and the compassionate mind	App	Clinician support as needed	Strengthen emotional literacy and tolerance through compassionate framing
4	Compassionate thinking	App	Clinician support as needed	Support cognitive coping and reduce self-criticism via compassionate self-talk
5	Preparing for trauma narrative with compassion	App	Clinician support as needed	Build emotional scaffolding and readiness for trauma work

a Modules were released sequentially according to the planned weekly schedule. Participants could complete each module flexibly within the assigned week but could not access subsequent modules before their scheduled release. Adolescents accessed the intervention through the app, whereas caregivers received parallel content through PDFs and QR-linked materials because the caregiver app was not yet available.

#### Platform and Technical Framework

The stabilization module was delivered via a web-based test environment of the Amni platform [47], accessed through secure authentication. Although implemented in a controlled test environment for research purposes, the underlying platform is a Medical Device Regulation (MDR)–compliant, CE-marked medical device. The study used this test environment to enable early-stage evaluation of usability, acceptability, and technical functionality. All data were transmitted over encrypted channels and stored on secure regional health care servers in compliance with the GDPR.

A full production version of the Amni app, with additional features such as integrated clinician dashboards, automated reminders, and enhanced technical stability, is currently under development and will be used in the subsequent pilot RCT. Insights from the present formative study will inform this production version.

Videoconferencing sessions were conducted using GDPR-compliant telehealth systems (Visiba Care and Platform24), and technical support was available for login, navigation, and other digital challenges.

#### Caregiver Version

A caregiver version of the app was developed to parallel the adolescent modules and included additional content on caregiver self-care, emotion regulation, and compassion-based strategies intended to support caregivers in their role throughout treatment. For the present study, the caregiver app had not yet been released in its production format, so caregivers accessed the materials via PDFs and QR-linked content. In the full implementation of bTF-CBT-C, caregivers will use a dedicated app that mirrors the adolescent modules and supports coordinated regulation strategies and a shared therapeutic understanding. Consequently, caregiver experiences reported in the present study reflect engagement with parallel intervention materials delivered through PDFs and QR-linked content rather than through the planned caregiver app.

Figure 1 shows screenshots illustrating core app functions.

**Figure 1.**
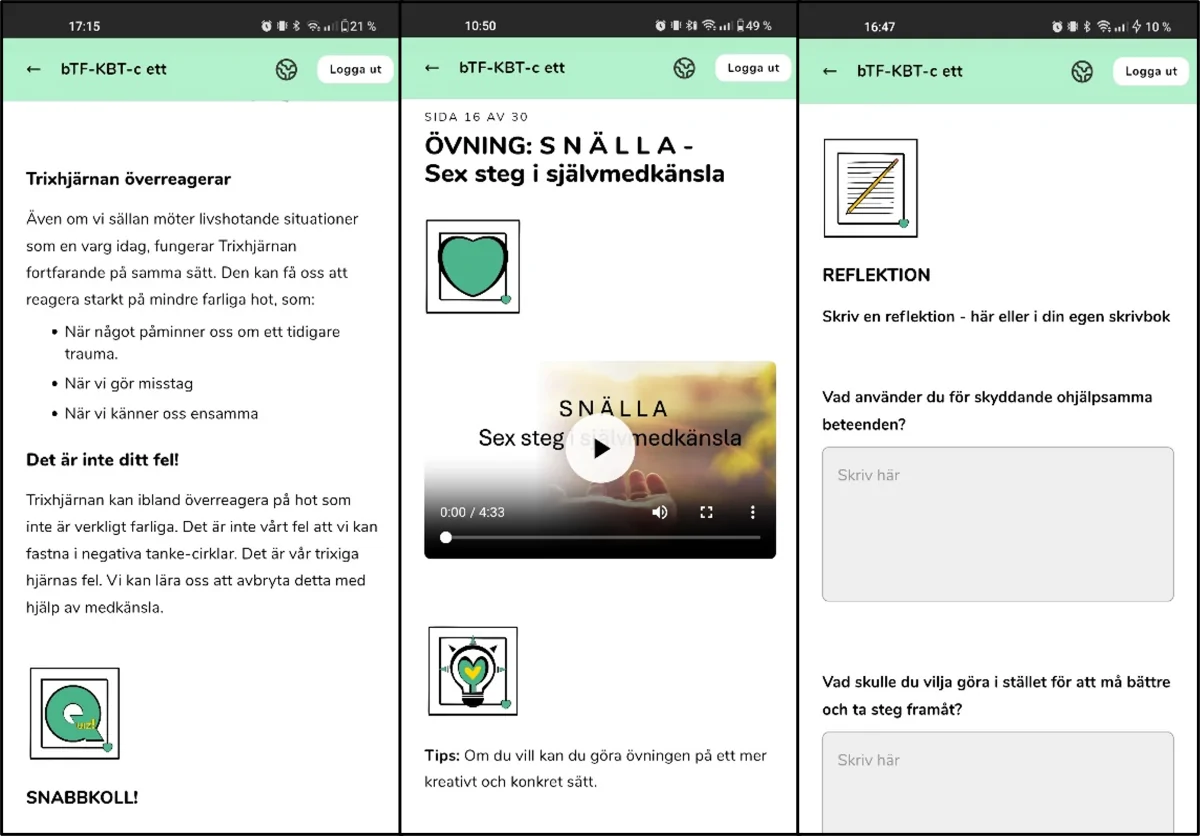
Screenshots from the adolescent version of the blended trauma-focused cognitive behavioral therapy with compassion (bTF-CBT-C) stabilization app used in a qualitative pretrial study of adolescents with posttraumatic stress disorder (PTSD) conducted at 2 child and adolescent psychiatry (CAP) clinics in northern Sweden (June-October 2025). The screenshots illustrate psychoeducation about self-critical thinking and the threat system, a guided compassion exercise (“SNÄLLA [Kind]—6 steps of self-compassion”), and a reflective writing task. The original interface language was Swedish.

### Procedure of Intervention Implementation

The intervention was delivered in a structured, blended format that combined app-based work with clinician support. After enrollment, adolescents and caregivers completed baseline self-report measures. Participants then received access to the introductory module of the stabilization phase, marking the official start of the 5-week app stabilization period. Adolescents accessed the app version of the introductory module, while caregivers received the corresponding materials in PDF format via secure delivery, as the caregiver app was not yet available in production.

After completing the introductory module, each family attended an in-person session at their local CAP clinic. During this visit, a licensed psychologist or psychotherapist introduced the bTF-CBT-C model, demonstrated app navigation, reviewed safety routines, and addressed initial questions. Adolescents also received a small “safety box” containing pens, colored pencils, a notepad, and a fidget tool to support emotion regulation during home-based practice.

To promote early engagement during the initial phase of app use, clinicians scheduled a follow-up videoconference about 1 week after the in-person introductory session, when adolescents had begun working with module 1. This meeting provided an opportunity to review experiences, clarify instructions, and address technical or emotional challenges. Throughout the 5-week stabilization period, clinicians remained available through brief in-app messages and, when required, telephone contact. Although no fixed schedule for messaging was prescribed, clinicians were encouraged to review participant progress regularly, typically at least once per week, and provide reminders, encouragement, clarification, or support as needed.

During the stabilization phase, the app-based intervention constituted the active treatment component while clinician involvement focused on support, monitoring, and preparation for the subsequent trauma-focused phases of TF-CBT. Safety monitoring followed routine CAP procedures and included opportunities to discuss emotional reactions and review concerns that arose during stabilization. Clinicians also participated in regular support meetings to discuss procedural questions; monitor adherence to safety routines; and identify potential adverse events, technical issues, and logistical challenges.

After completing the 5-week stabilization phase, adolescents transitioned to the trauma processing and integration phases of therapist-delivered TF-CBT with the same clinician at their local CAP clinic. No trauma processing was conducted during the stabilization phase, which was designed to prepare adolescents and caregivers for subsequent trauma-focused work.

### Data Analysis

Data were analyzed using the reflexive thematic analysis approach described in the *Methods* section. The analytic process began with repeated readings of all qualitative materials to achieve familiarity, followed by generating initial inductive codes across adolescent interviews, caregiver interviews, and clinician survey responses. Coding was conducted by LW and NN using OpenCode (version 4.03) and supplemented with manual coding using printed excerpts and paper slips to support reflexive engagement with the data.

Codes were then iteratively developed into initial theme candidates by examining patterned meanings across the integrated dataset. Through repeated movement between data, codes, and analytic memos, themes were reviewed, refined, and rearticulated. Theme development was led by LW in ongoing reflection and discussion with NN and ID. Final themes were defined based on their contribution to understanding acceptability, feasibility, and refinement needs prior to the pilot RCT.

Throughout the analysis, all authors reflected on how their professional backgrounds, experiences, and involvement in the project might shape interpretation of the data. LW is a licensed psychotherapist and doctoral student who led the present study. ID is a clinical psychologist, senior researcher (PhD), and principal investigator of the broader research project. LW and ID were involved in developing the intervention and had clinical experience in TF-CBT and CFT, which informed their understanding of the study context. The team developed interpretations through ongoing discussions to critically examine assumptions, challenge emerging interpretations, and consider alternative explanations. Consistent with reflexive thematic analysis, these discussions were intended to deepen interpretive understanding rather than achieve coding consensus.

## Results

The analysis yielded 1 overarching theme and 5 interconnected themes that describe how adolescents, caregivers, and clinicians experienced the app-based stabilization phase of bTF-CBT-C.

### Overarching Theme: Navigating Digital Stabilization Within Shifting Conditions

#### Overview

The overarching theme portrays the stabilization phase as a dynamic, nonlinear process continuously shaped by fluctuations in emotional capacity, cognitive load, relational dynamics, and logistical conditions. Participants’ ability to engage fluctuated, with digital work sometimes supported and at other times constrained by everyday life. Across groups, the app provided structure, grounding, and moments of preparedness for upcoming trauma-focused work, yet engagement was highly sensitive to day-to-day variations in emotional and practical conditions.

Within this overarching theme, five interconnected themes describe distinct, but related dimensions of experience: (1) the tension between support and strain, (2) when clarity meets cognitive limits, (3) autonomy in relational uncertainty, (4) when logistical fragility disrupts momentum, and (5) grounding as a bridge to trauma-focused work. See Figure 2 for an overview of these themes.

**Figure 2.**
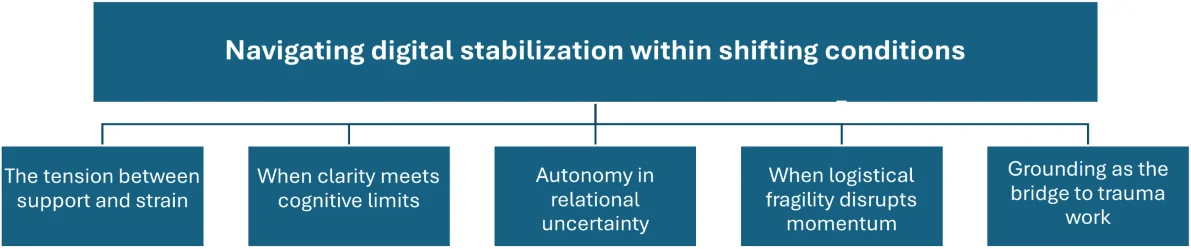
Overarching theme and 5 interrelated themes describing experiences of app-based stabilization within blended trauma-focused cognitive behavioral therapy with compassion (bTF-CBT-C) among adolescents with posttraumatic stress disorder (PTSD), caregivers, and clinicians in a qualitative pretrial study conducted at 2 child and adolescent psychiatry (CAP) clinics in northern Sweden (June-October 2025).

#### Theme 1. The tension between support and strain

This theme captures the ongoing tension between the intervention’s supportive qualities and the strains of everyday life, a dynamic that shaped when, how, and to what extent adolescents and caregivers could engage with the stabilization phase. Flexibility, grounding practices, and structure were consistently valued, yet their benefits were continually pulled against emotional fatigue, stress, neurodivergent attention patterns, and practical limitations in family life.

Adolescents described engaging at moments when internal capacity aligned:

*With ADHD, nothing becomes regular…the good thing was that I could do it when my brain was calmer.*

[Adolescent 2]

Even when motivation was high, strain reentered the process, making reminders essential:

*It was hard to remember sometimes…I needed reminders from my therapist and my dad.*

[Adolescent 3]

Caregivers described a similar push-and-pull. Their willingness to support was often strong, while their capacity to enact that support fluctuated:

*My willingness wasn’t the problem…my life situation was.*

[Caregiver 1].

Clinicians also highlighted how families’ fluctuating capacity shaped feasibility. One therapist noted that the intervention was difficult to sustain when family routines faltered, or caregiver support was inconsistent:

*When the parent doesn’t remember to do the exercises themselves or set aside time for the app, it also becomes difficult for the young person.*

[Therapist 1]

Across groups, feasibility served as a resource-dependent balancing act, in which support and strain coexisted rather than canceling each other out. Engagement fluctuated with mood, energy, attention-deficit/hyperactivity disorder–related variability, work schedules, and caregiver strain. These dynamics highlight the importance of predictable reminders, flexible pacing, and clear caregiver guidance to sustain motivation during the stabilization phase.

#### Theme 2. When Clarity Meets Cognitive Limits

This theme captures the tension between the app’s pedagogical clarity and the cognitive limits imposed by fatigue, stress, neurodivergence, and daily overload. Participants consistently described the material as clear and accessible. Videos, visuals, and structured psychoeducation made complex trauma concepts more easily graspable, yet the ability to absorb and use this clarity depended on moment-to-moment cognitive bandwidth. Adolescents highlighted how multimedia elements helped with understanding and motivation:

*After the film, you could do a little quiz…it made me more motivated.*

[Adolescent 1]

*Sometimes you don’t have the energy to read; then you can just listen.*

[Adolescent 2]

The flexibility to switch between text and video helped adolescents stay engaged when attention fluctuated. But even well-designed content could exceed available cognitive resources during tired or stressful periods:

*The chapters were a bit long…I lost interest and got irritated.*

[Adolescent 2]

Caregivers also valued this clarity. One caregiver described how a visual metaphor became so meaningful that she tore the “trick brain” page out and put it on the refrigerator to keep it visible. Yet, clarity alone was not enough when cognitive capacity was low. Information-dense chapters or longer text passages became overwhelming late in the day or during stressful periods:

*Quite a lot to read…especially in the evenings when you’re already tired.*

[Caregiver 2]

Participants also described how features intended to support pacing sometimes broke cognitive flow. Mid-chapter pause pages, for example, could interrupt engagement just when focus had finally ramped up:

*I worked a lot when I was in there…then a pause page popped up…it was a bit annoying in the middle of everything.*

[Adolescent 3]

From a clinical perspective, therapists observed that while the content was conceptually clear, it was not always cognitively easy to take in:

*Some chapters required more stamina than they had…even if the content was good.*

[Therapist 3]

These accounts demonstrate that even clear, well-designed content can overwhelm limited cognitive capacity, especially after school, late at night, or during emotional stress. This underscores the need for shorter chapters, less text, more visual formats, and pacing aligned with adolescents’ and caregivers’ changing mental bandwidth.

#### Theme 3. Autonomy in Relational Uncertainty

This theme describes how adolescents’ need for autonomy interacted with caregivers’ uncertainty about how, when, or whether to support them. The digital format appeared to increase privacy and a sense of control, making engagement feel manageable for adolescents who preferred independence. For some, this independence was a necessary condition for participation:

*We sat at the same table but worked separately.*

[Adolescent 1]

Clinicians recognized the same pattern from a therapeutic perspective, noting that several adolescents were more willing or able to engage when they could work independently, without feeling observed or pressured by others. Their accounts indicated that autonomy facilitated participation among youth who needed distance to remain steady and engaged.

At the same time, adolescents’ autonomy created relational uncertainty for caregivers. Many wanted to help but were unsure how to do so without intruding:

*She didn’t want me there…I didn’t know how to help.*

[Caregiver 1]

Despite this tension, the digital format also appeared to create openings for connection and shared understanding. Adolescents described moments of increased emotional expression:

*I’ve dared to talk to my parents about how I feel.*

[Adolescent 2]

Caregivers similarly noticed rare moments of closeness emerging:

*She gave me a hug the other day…she never does that.*

[Caregiver 2]

Clinicians described this dynamic as a delicate balance for families. Too much involvement could feel overwhelming, whereas too little risked leaving adolescents without needed scaffolding. Families often struggled to maintain this balance, and clinicians emphasized that relational sensitivity and flexible coordination were essential to keeping the work moving forward in a supportive way.

Across groups, autonomy and relational support are interconnected processes that require ongoing negotiation. These dynamics highlight the importance of clear caregiver guidance and structured joint activities in the pilot RCT, which can help turn relational willingness into practical action without compromising adolescents’ sense of control.

#### Theme 4. When Logistical Fragility Disrupts Momentum

This theme captures how the therapeutic value of the digital stabilization phase depended on the stability of the surrounding logistical and technical systems. Even when adolescents and caregivers were motivated and emotionally ready, small logistical disruptions, such as login friction, inconsistent notifications, onboarding complexity, or platform glitches, could break momentum and interrupt therapeutic flow.

Adolescents described how early login difficulties or unexpected hurdles stalled engagement:

*It lagged sometimes…then I had to wait, and I didn’t want to go back.*

[Adolescent 3]

Caregivers encountered similar friction, amplified by fatigue and time pressure when switching between PDFs, links, or codes:

*It didn’t work with BankID…then I forgot the password…it became a barrier.*

[Caregiver 1]

Clinicians described how technical and logistical breakdowns, rather than emotional or relational challenges, often demanded additional work on their part. When families lost access, encountered login loops, or were unable to navigate PDFs and links, clinicians stepped in to restore access, resend information, or manually unlock content. These practical fixes took time and interrupted the therapeutic flow, highlighting how small points of friction in the digital infrastructure could create disproportionate disruption during an already vulnerable phase.

At the system level, participants noted that such disruptions could erode confidence, disrupt grounding practices, and derail routines that families were only beginning to establish. As one clinician expressed:

*The positive feedback should ideally reach the youth immediately.*

[Therapist 1]

Across groups, engagement relied not only on emotional, cognitive, and relational resources but also on stable, predictable, low-friction pathways into digital intervention. When logistical fragility surfaced, therapeutic momentum was easily lost, underscoring the need for streamlined onboarding, reliable notifications, simplified login flows (eg, consistent BankID), and an integrated caregiver interface before conducting the pilot RCT.

#### Theme 5. Grounding as the Bridge to Trauma Work

This theme describes how grounding practices, compassion-based strategies, and clear psychoeducation created an emotional foundation that prepared adolescents, caregivers, and clinicians for the trauma-focused phase. Across groups, participants highlighted how the stabilization work increased safety, emotional coherence, and capacity to tolerate distress, which allowed adolescents to approach trauma content with greater steadiness.

Adolescents frequently described grounding practices as *portable* tools they could rely on in moments of overwhelm, including in school or other demanding settings:

*I use the safe place at school when things get overwhelming.*

[Adolescent 3]

They also described a growing sense of emotional insight:

*I understand my feelings in a different way…why I react as I do.*

[Adolescent 2]

Caregivers emphasized that working with the compassion-based models each week, including mapping both their own and their adolescent’s threat-drive-soothing systems, helped them interpret emotional shifts more clearly:

*The threat system got smaller and smaller…it made sense in a new way.*

[Caregiver 1]

Some caregivers also experienced shifts in their own emotional processing:

*Many feelings came up…but I also started being a bit kinder to myself.*

[Caregiver 2]

Clinicians observed that adolescents who practiced grounding entered sessions more regulated and able to remain within a tolerable arousal window during early trauma-related discussions:

*She came in with a different understanding of herself.*

[Therapist 3]

This increased stability made it easier to transition into narrative work. For clinicians, grounding created predictable emotional baselines that supported therapeutic pacing and reduced early-phase ruptures.

Taken together, these accounts suggest that grounding, compassion practices, and psychoeducation may have contributed to perceived emotional readiness for trauma-focused therapy. This readiness did not necessarily arise from resolving the relational uncertainties described in theme 3, but rather seemed linked to strengthened regulation, enhanced self-understanding, and the development of a shared conceptual language. In this way, participants described the stabilization phase as functioning as a bridge that helped prepare adolescents, caregivers, and clinicians for the cognitive, emotional, and physiological demands of trauma processing.

### Summary of Results

Across adolescents, caregivers, and clinicians, the app-based stabilization phase was experienced as a dynamic process shaped by shifting emotional, cognitive, relational, and logistical conditions. Participants consistently described the intervention as meaningful, grounding, and clarifying, yet effortful to sustain within the realities of everyday life. Engagement depended not only on motivation but on the fluctuating resources that families could mobilize from week to week.

The 5 themes together illustrate how these shifting conditions played out. Adolescents and caregivers navigated the tension between support and strain in which moments of capacity enabled engagement, while fatigue, stress, and life pressures pulled against it. Even when the material was clear and accessible, pedagogical clarity sometimes collided with limited cognitive bandwidth, particularly after school or during periods of overload. Engagement also unfolded amid relational uncertainty, as adolescents sought autonomy while caregivers struggled to gage when and how to be involved.

Beyond these interpersonal and intrapersonal dynamics, logistical fragility often disrupted momentum, with small technical or procedural barriers (eg, login issues, missed notifications, unstable access) breaking flow even when motivation and readiness were present. Yet, despite these challenges, the stabilization phase seemed to foster emotional grounding and readiness for trauma-focused work, giving adolescents practical regulation tools, deeper emotional insight, and a shared language that supported the transition into therapist-delivered TF-CBT.

Taken together, these findings suggest that perceived feasibility was shaped by emotional, cognitive, relational, and logistical conditions. Aligning content design, technical stability, and clear relational guidance will therefore be essential to optimizing acceptability and feasibility in the upcoming pilot RCT.

### Refinements for the Pilot RCT Based on the Reflexive Thematic Analysis

To translate the thematic insights into practical improvements for the upcoming pilot RCT, we developed a set of refinements directly informed by the 5 themes. These refinements capture the lived constraints, cognitive and emotional capacities, relational patterns, and logistical realities described by adolescents, caregivers, and clinicians. By linking challenges to actionable adjustments, Table 2 summarizes how the intervention can be improved to boost feasibility and acceptability and better match families’ real-world situations.

These refinements show how thematic insights can directly guide intervention optimization before the pilot RCT. Overall, they emphasize that feasibility depends not only on content quality but also on the match between therapeutic material, users’ cognitive and emotional resources, family system dynamics, and the practical conditions for delivering digital care.

**Table 2.** Proposed refinements to the blended trauma-focused cognitive behavioral therapy with compassion (bTF-CBT-C) stabilization phase for the pilot randomized controlled trial (RCT) based on reflexive thematic analysis of adolescents‘, caregivers,’ and clinicians’ experiences in a qualitative pretrial study conducted at 2 child and adolescent psychiatry (CAP) clinics in northern Sweden (June-October 2025).

Theme	Identified challenges	Refinements needed for pilot RCT	How refinement supports feasibility and acceptability
The tension between support and strain	Competing demands and fatigue Need for reminders Variable caregiver support Attentional variability	Flexible pacing Automated reminders Weekly microgoals Progress feedback	Supports engagement despite fluctuating emotional and practical demands
When clarity meets cognitive limits	Long/text-heavy content Attentional challenges High cognitive load Underused visual supports	Shorter chapters More visual and interactive content Improved pacing Simplified exercises	Improves cognitive accessibility and reduces overload
Autonomy in relational uncertainty	Preference for autonomy Unclear caregiver role Variable family readiness Inconsistent collaboration	Joint chapter activities Clear caregiver guidance Support for adolescent autonomy Dedicated caregiver app Family reflection prompts	Strengthens caregiver support while respecting adolescent autonomy
When logistical fragility disrupts momentum	Login barriers Navigation difficulties Inconsistent reminders Time-consuming onboarding	Simplified login Reliable notifications Clinician onboarding guide In-app user walkthrough	Reduces technical barriers and supports continued engagement
Grounding as the bridge to trauma work	Distress during exercises Need for emotional safety Need for shared understanding Importance of clear rationale	Early normalization and safety information Quick-access grounding tools Explicit links to trauma work Shared family summaries	Strengthens emotional readiness for trauma-focused work

## Discussion

### Principal Findings

This qualitative formative study explored how adolescents with PTSD, their caregivers, and clinicians experienced a 5-week app-based stabilization phase delivered within the bTF-CBT-C intervention. In our analysis, we conceptualized digital stabilization as a dynamic rather than linear process, reflecting the overarching theme. Digital stabilization was identified as both meaningful and effortful, shaped by shifting emotional capacity, cognitive bandwidth, relational dynamics, and day-to-day practical conditions. Feasibility appeared to depend more on adolescents’ and families’ fluctuating access to internal and external resources than on motivation alone. Given the formative design and small sample, these findings should be interpreted as preliminary and specific to the study context.

The findings align with research indicating that digital and blended trauma-focused approaches can improve accessibility and reduce logistical and emotional barriers in youth trauma treatment [20,21,48]. Prior studies show that when digital formats fit adolescents’ needs, they can support engagement and emotional insight [20,22,36,49,50]. Our results extend this literature by clarifying how these engagement dynamics unfold within a digital stabilization phase, a component that has received limited empirical attention in digital trauma interventions.

Beyond these observations, our findings align with longstanding evidence from youth psychotherapy showing that engagement fluctuates with emotional regulation capacity, attentional variability, and family system load rather than treatment interest alone [51]. Similar fluctuation patterns are reported in digital youth mental health, where daily cognitive and emotional rhythms shape engagement [2]. This research helps contextualize how such dynamics operate during digital stabilization preceding trauma-focused work, a step rarely examined in prior studies.

Consistent with affect-regulation models, adolescents’ engagement fluctuated with arousal levels, cognitive load, and attentional variability [11,52]. Even structured and multimodal content was experienced as demanding during periods of fatigue or emotional overload. These fluctuations may be particularly pronounced when PTSD co-occurs with neurodivergent attentional profiles [53,54]. Within a CFT framework, these patterns align with shifts between threat and soothing systems [26], underscoring that cognitive and emotional availability (not motivational readiness) may be important for feasibility.

PTSD-related avoidance may also have contributed to some of the engagement patterns observed in the present study. Avoidance is a core feature of PTSD and may influence willingness to engage with trauma-related material, emotional reflection, or therapeutic exercises [4,11,52]. Consequently, fluctuations in participation, reduced engagement, or difficulty completing modules may reflect not only practical barriers, cognitive load, or usability challenges but also adaptive attempts to avoid distressing thoughts, emotions, or trauma-related content. Future studies should examine more explicitly how avoidance symptoms interact with engagement during digital stabilization and trauma-focused treatment.

These findings also resonate with qualitative work in Swedish CAP settings, where adolescents expressed a preference for blended formats that increase accessibility, reduce barriers, and offer stabilizing support between sessions [25]. At the same time, much of the existing evidence for digital youth interventions is based on samples with mild-to-moderate difficulties, with clinically complex populations often underrepresented in trials [1,55]. Engagement challenges and early dropouts are common in digital mental health [54,56], and therapists in our study noted that maintaining participation often required additional scaffolding such as reminders, troubleshooting, and brief check-ins. Against this backdrop, the meaningful engagement observed in our clinically complex CAP sample suggests that digital stabilization may be feasible for adolescents with clinically complex presentations who are often underrepresented in digital intervention research.

The findings also align with the literature, indicating that adolescents benefit from brief, visually supported, and flexible digital content [1,2]. Reviews similarly emphasize that short and adaptable modules are particularly important for adolescents with fluctuating attention [53,54]. Network meta-analytic evidence also indicates CBT-based digital interventions rank highly for youth anxiety, supporting structured, modular delivery in this population [57]. However, the observed tension between conceptual clarity and cognitive effort suggests that even well-designed material may be demanding during periods of low capacity, particularly for neurodivergent or highly burdened adolescents.

A notable contribution of this study concerns relational uncertainty around caregiver involvement in digital stabilization. Although caregivers accessed the content through PDFs and QR-linked materials rather than a dedicated app, their experiences highlighted how family routines, caregiver support, and relational dynamics influenced engagement throughout the stabilization phase.

While caregiver participation is known to enhance TF-CBT outcomes [58,59], less is known about how autonomy and support interact in digital modalities. Adolescents often preferred solitary work to avoid relational pressure, whereas caregivers struggled to provide nonintrusive support. CFT models can help explain this dynamic: threat-system activation and fears of misattunement can lead both youths and caregivers to withdraw or overcompensate [60,61]. These findings underline the need for explicit, developmentally attuned guidance for caregivers in blended stabilization, indicating that autonomy and support must be balanced.

Technical reliability also influenced feasibility. Even minor issues, such as login barriers or unstable notifications, affected adherence, mirroring findings across digital mental health research.

Despite a clinically complex sample characterized by PTSD, comorbidity, caregiver strain, and family adversity, adolescents engaged meaningfully with the stabilization module. This corresponds with emerging evidence that flexible, low-threshold digital formats can be feasible even for clinically complex youth [1,54,55], especially when supported through reminders, troubleshooting, and brief check-ins. In CAP contexts where emotional dysregulation, cognitive variability, and family strain are common, digital stabilization may therefore serve as a viable preparatory pathway.

Finally, the results are consistent with theoretical and clinical arguments for incorporating compassion-focused grounding in stabilization. CFT emphasizes soothing-system activation, shame reduction, and affect regulation as preparatory mechanisms for trauma work [26,62,63]. Participants described increased emotional insight, regulation capacity, and shared language for understanding emotional experiences, all of which align with the goals of TF-CBT’s stabilization. From a CFT perspective, compassion-based grounding may support emotional regulation and reduce threat-focused responding, which participants perceived as helpful when preparing for trauma-focused work [27,29]. These findings suggest that digital compassion-focused stabilization may serve as a bridge between early psychoeducation and later trauma-focused work.

### Implications for Intervention Design

The identified refinements highlight several principles for designing a feasible stabilization phase within blended TF-CBT-C: alignment with adolescents’ fluctuating emotional and cognitive rhythms, flexible pacing and lower information density, multimodal formats to support attentional variability, explicit caregiver guidance to balance autonomy and relational support, and reliable technical infrastructure. Grounding and compassion-based strategies should be explicitly connected to trauma-focused work to strengthen emotional readiness and shared understanding. Together, these principles underscore the broader design conditions required for digital components to function effectively within routine CAP practice.

### Strengths and Limitations

Strengths include the multistakeholder design (integrating the perspectives of adolescents, caregivers, and clinicians), a reflexive analytic approach, and an ecologically valid CAP context. Several limitations should also be considered. First, the sample was small and context-specific, and the findings should therefore be viewed as exploratory. The study was designed to inform intervention refinement prior to a pilot RCT and was not intended to provide evidence of clinical effectiveness or broader implementation feasibility. Second, all adolescent participants were girls. Experiences of digital engagement, autonomy, caregiver involvement, emotional expression, and treatment participation may differ among boys and gender-diverse youth. The findings may therefore not be transferable to broader adolescent populations and should be interpreted with caution regarding gender diversity. Third, the intervention was evaluated within a research test environment rather than the final production version of the platform. Some of the technical challenges reported in this study may therefore not reflect the functionality of the final platform.

Finally, caregivers accessed the stabilization content through PDFs and QR-linked materials because the caregiver app was not yet available. Their experiences therefore reflect the caregiver intervention content delivered through an alternative format rather than the planned caregiver app.

### Implications for Future Trials and Implementation

For a pilot RCT to yield interpretable feasibility data, platform reliability, caregiver integration, and pacing flexibility should be prioritized. Regional conditions, such as long distances and uneven availability of specialists, necessitate attention to implementation outcomes, such as workflow compatibility and notification efficacy. Offline access, reduced data load, and low-bandwidth optimization will be important for equitable participation. Mixed-methods process evaluation can clarify dose-response patterns, identify bottlenecks, and inform iterative improvement.

### Conclusions

This study suggests that an app-based stabilization phase for adolescents, together with parallel caregiver materials, within bTF-CBT-C may be acceptable and potentially feasible. Participants identified both strengths of the intervention and areas requiring refinement, particularly related to technical functionality, cognitive accessibility, and caregiver support. The findings highlight candidate refinements for evaluation in the pilot RCT and offer preliminary guidance for optimizing the intervention and its implementation. These findings are preliminary and should be interpreted within the context of this small formative qualitative study.

## Supplementary material

10.2196/95633 Checklist 1 Reflexive Thematic Analysis Reporting Guidelines.

## Abbreviations

bTF-CBT-C: blended trauma-focused cognitive behavioral therapy with compassion
CAP: child and adolescent psychiatry
CFT: compassion focused therapy
DSM-5: Diagnostic and Statistical Manual of Mental Disorders, Fifth Edition
GDPR: General Data Protection Regulation
MDR: Medical Device Regulation
PTSD: posttraumatic stress disorder
RCT: randomized controlled trial
RTA: reflexive thematic analysis
RTARG: Reflexive Thematic Analysis Reporting Guidelines
TF-CBT: trauma-focused cognitive behavioral therapy
