# Supplementary material for: App-Based Stabilization Phase Within Blended Trauma-Focused Cognitive Behavioral Therapy With Compassion for Adolescents With Posttraumatic Stress Disorder: Formative Qualitative Study
Source: JMIR Form Res. 2026 Oct 15;10:e95633. doi: 10.2196/95633 (PMC13671487; doi:10.2196/95633)
Supplement: Supplementary file 1 [file formative-v10-e95633-s001.pdf]

Reflexive Thematic Analysis Reporting Guidelines (RTARG)

Manuscript title:
App-Based Stabilization for Adolescents with PTSD: A Formative Qualitative Study to Inform a Pilot RCT of Blended TF-CBT with Compassion

Authors: Linda Wallin et al.

Reporting guidance: Reporting of this study was informed by the Reflexive Thematic Analysis Reporting Guidelines (RTARG) proposed by Braun and Clarke (2024)[1]. The table below outlines how the manuscript addressed key RTARG recommendations.

RTARG Guideline	Location in Manuscript	How Addressed
Study purpose and research questions are clearly stated	Introduction	Explicit study aim and research question provided.
Rationale for using reflexive thematic analysis is provided	Data Analysis	Reflexive thematic analysis following Braun and Clarke is described and justified as appropriate for the study aims.
Theoretical and conceptual foundations of the study are described	Introduction; Data Analysis	The study context, intervention rationale, and analytic approach are described.
Epistemological and/or ontological assumptions are identified where relevant	Data Analysis	Reflexive thematic analysis is described following Braun and Clarke. Interpretive assumptions underpinning the analysis are acknowledged where relevant.
Researcher positioning and reflexivity are described	Data Analysis	Research team backgrounds, professional experience, and reflexive discussions during analysis are described.
Participant recruitment and sampling are described	Participants	Recruitment procedures, inclusion criteria, and participant characteristics are reported.
Context and setting are adequately described	Methods	The study context, intervention, and setting are described.
Data collection methods are described in sufficient detail	Procedure	Interview procedures, recording, transcription, and interview guide are described.
Reflexive thematic analysis procedures are described clearly	Data Analysis	Analytic procedures are described following Braun and Clarke's reflexive thematic analysis approach.
The role of the researcher(s) in	Data Analysis	Researchers’ active role in coding,

analysis is explained		interpretation, and theme development is described.
Coding procedures are described appropriately for reflexive thematic analysis	Data Analysis	Coding is described as an iterative and interpretive process.
Theme development is described as an active and interpretive process	Data Analysis	Themes were developed through iterative engagement with the data and ongoing team discussions.
Themes are presented as patterns of shared meaning rather than topic summaries	Results	Themes are presented as interpretive patterns of shared meaning supported by participant accounts.
A thematic map, figure, or table is provided where appropriate	Figure 2	A thematic map illustrating the overarching theme and interconnected themes is provided.
Themes are supported by relevant data extracts	Results	Each theme is illustrated with participant quotations.
Analytic claims are supported by the data presented	Results	Interpretations are supported by multiple participant quotations.
Consistency between theoretical framework, methods, analysis, and interpretation is demonstrated	Throughout manuscript	The study demonstrates consistency between the aims, reflexive thematic analysis approach, findings, and interpretation.
Strengths and limitations of the study are discussed	Discussion	Study strengths and limitations are discussed.
Implications of the findings are discussed	Discussion	Clinical and research implications are discussed.
Reporting demonstrates reflexive openness, clarity, and methodological coherence	Throughout manuscript	Reporting was reviewed against RTARG and revised to improve clarity, reflexive openness, and methodological coherence

1. Braun V, Clarke V. Supporting best practice in reflexive thematic analysis reporting in Palliative Medicine: A review of published research and introduction to the Reflexive Thematic Analysis Reporting Guidelines (RTARG). Palliat Med. 2024 Jun;38(6):608-16. PMID: 38469804. doi: 10.1177/02692163241234800.
